# An Attachment Perspective on Favorite Media Figures

**DOI:** 10.1177/00332941211002142

**Published:** 2021-03-25

**Authors:** A. Luke MacNeill, Enrico DiTommaso

**Affiliations:** Department of Psychology, University of New Brunswick, Canada

**Keywords:** Attachment theory, attachment anxiety, parasocial relationships, media figures

## Abstract

Anxiously attached individuals tend to report stronger parasocial relationships with their favorite media figures than people with other attachment orientations. Researchers have suggested that these individuals may be inclined to see their favorite media figures as safe and secure attachment figures. The purpose of the current study was to evaluate this possibility by assessing the qualities of people’s favorite media figures, particularly within a television context. A sample of 200 online participants filled out an attachment measure, reported their favorite television figure, and rated several aspects of the television figure’s personality. It was expected that anxiously attached individuals would be drawn to figures that are high in warmth, emotional stability, and sensitivity. Instead, results showed that these individuals preferred figures with greater anxious and insecure characteristics. These results suggest that anxiously attached individuals may not see their favorite media figures as safe and secure attachment figures as previously theorized. Exploratory analyses failed to show significant effects for the second attachment dimension, attachment avoidance, or for the interaction between anxiety and avoidance.

## Introduction

People often form intimate bonds with celebrities and fictional characters they encounter in the media (Hartmann, 2016; Rosaen & Dibble, 2016; Schramm & Hartmann, 2008). Although these “parasocial” relationships are one-sided and nonreciprocal in nature, they appear to be closely related to the social relationships that people develop with real, flesh-and-blood humans. For instance, many of the processes that underlie the formation and maintenance of social relationships are also relevant for parasocial relationships (Kassing & Sanderson, 2009; Rubin & McHugh, 1987; Tsao, 1996). In addition, parasocial relationships can provide individuals with feelings of belonging and help fill the void that they experience when they are having problems meeting their social needs (Derrick et al., 2009; Gardner et al., 2005). Based on these and similar findings, researchers have suggested that theories of social relationships may be a useful means of advancing our understanding of relationships with media figures (e.g., Eyal & Dailey, 2012; Rubin & McHugh, 1987). One such theory that has received an increasing amount of attention in recent years is attachment theory.

Attachment theory arose from the work of British psychiatrist and psychoanalyst John Bowlby (1969, 1973). Bowlby proposed that humans have an innate attachment behavioral system that guides their functioning in close relationships. In times of threat or danger, this system motivates people to seek out significant others who can relieve their distress. These latter individuals are known as attachment figures. Warm, consistent, and responsive attachment figures will alleviate distress and provide individuals with a sense of security (Mikulincer & Shaver, 2007). Unavailable, rejecting, or inconsistently responsive attachment figures will fail to mitigate distress and trigger feelings of insecurity.

Early experiences with attachment figures become internalized as *working models*, mental representations of the self and close others that shape a person’s expectations regarding their close relationships (Bowlby, 1969, 1973). These working models manifest in a characteristic attachment orientation that affects an individual's behavior over the lifespan. Adult attachment orientations are often conceptualized along two dimensions: attachment anxiety and attachment avoidance (Brennan et al., 1998). People with high levels of attachment anxiety are preoccupied with their close relationships and have an excessive fear of being rejected or abandoned by their relationship partners. They tend to exhibit hyperactivating attachment strategies that involve energetic and excessive reassurance seeking. People with high levels of attachment avoidance are uncomfortable with emotional intimacy and demonstrate high levels of independence and self-reliance. They tend to exhibit deactivating attachment strategies that involve the suppression of attachment-related thoughts and emotions. People who are low in both attachment anxiety and attachment avoidance are said to be high in attachment security.

Attachment orientations have implications beyond one’s close social partners. For instance, individuals with high levels of attachment anxiety tend to form stronger parasocial relationships with their favorite media figures than people with other attachment orientations (Cohen, 2004; Cole & Leets, 1999; Jin & Kim, 2015; Rosaen & Dibble, 2016; Theran et al., 2010). To explain this finding, researchers have looked to the functioning of anxiously attached individuals in their conventional attachment relationships. Anxiously attached individuals are inclined to have a negative view of themselves and an idealized view of close others, a combination of views that can trigger feelings of insecurity and elicit clingy or dependent behavior (Bartholomew & Horowitz, 1991; Mikulincer & Shaver, 2007). If close relationship partners fail to provide high levels of responsivity and support, then anxiously attached individuals might feel rejected and experience low levels of relationship satisfaction. Therefore, these individuals may come to see media figures as surrogate attachment figures that are more accessible, reliable, and safe (i.e., free from rejection or disapproval) than close relationship partners in real life. In other words, media figures may offer these individuals the chance for a secure connection (Cole & Leets, 1999; Greenwood et al., 2008; Theran et al., 2010).

To our knowledge, the notion that anxiously attached individuals see media figures as safe and secure attachment figures has received no empirical attention to date. However, it could be assessed by determining the qualities of people’s favorite media figures. If the surrogacy hypothesis were true, then one would expect anxiously attached individuals to be drawn to media figures that are high in warmth, emotional stability, and sensitivity, those same qualities that they are seeking (and likely not finding, to their satisfaction at least) in their real-life attachment figures.

*Hypothesis 1:* Individuals with high levels of attachment anxiety have favorite media figures that are high in warmth, emotional stability, and sensitivity.

One might wonder whether individuals with high levels of attachment avoidance are drawn to media figures that possess certain qualities. Unlike anxiously attached individuals, people with high levels of attachment avoidance do not appear to have particularly strong parasocial relationships with their favorite media figures (Cohen, 2004; Cole & Leets, 1999; Jin & Kim, 2015; Rosaen & Dibble, 2016; Theran et al., 2010). It seems unlikely that they seek out surrogate attachment figures in the media as a means of compensating for a lack of attachment security in their real lives. Taking this into consideration, it is doubtful that their favorite media figures are overly warm, emotionally stable, or sensitive. It remains to be seen whether they prefer media figures with other qualities. For instance, given their somewhat standoffish and aloof personalities, they may be drawn to media figures that are low in warmth and sensitivity and high in self-reliance. However, this possibility is speculative.

*Research Question 1:* Do individuals with high levels of attachment avoidance have favorite media figures that possess certain qualities?

Studies in this area tend to consider attachment anxiety and attachment avoidance separately, but it should be noted that these two dimensions can interact with one another to create variations in attachment orientations. For instance, some people report high levels of attachment anxiety and low levels of attachment avoidance, whereas other people show the opposite pattern. Still others report high levels or low levels of both dimensions. It is currently unclear whether these dimensions interact to affect media figure preferences, and if so, what the specific pattern of results might be. Although it is expected that high levels of attachment anxiety will be related to a preference for safe and secure media figures, the uncertainty surrounding the avoidance dimension makes it difficult to predict how it might interact with anxiety to impact media figure preferences.

*Research Question 2:* Is the interaction between attachment anxiety and attachment avoidance related to favorite media figure qualities?

The purpose of the current study was to learn more about how attachment dimensions are related to media figure preferences. This study focused on television figures specifically, as past research in this area has concentrated primarily on this type of media (e.g., Cohen, 2004; Cole & Leets, 1999; Jin & Kim, 2015; Rosaen & Dibble, 2016).

## Method

### Participants

Participants were recruited through the crowdsourcing Internet marketplace Amazon Mechanical Turk (MTurk). Participation was restricted to residents of the United States and Canada who had a successful completion rate of 97% or above for their MTurk tasks. A total of 288 individuals completed the study. Participants were dropped from the analysis if they failed attention questions embedded within the questionnaire (*N* = 22), if they provided suspicious or incomprehensible answers (e.g., bot responses) (*N* = 25), if they selected a media figure that did not appear on television (*N* = 27), or if they were statistical outliers that provided extreme or unusual scores (*N* = 14). This left a final sample of 200 participants. The sample consisted of 89 males and 111 females. Participants had a mean age of 44.06 years (*SD* = 13.60). The sample was largely White (79%), with the rest of the sample identifying as Black or of African descent (8%), Asian or Pacific Islander (7.5%), or another race or ethnicity (5.5%). Participants were offered a monetary incentive of 50 cents American for their participation in the study.

### Measures

A demographic questionnaire included questions regarding participant age, gender, and race/ethnicity.^1^ Other instruments are described below.

*Attachment orientations.* Attachment orientations were assessed with a 36-item scale from Brennan et al. (1998). This scale consists of two subscales, one to measure attachment anxiety and another to measure attachment avoidance. Sample items from this scale include “I worry about being rejected or abandoned” for attachment anxiety and “I prefer not to show others how I feel deep down” for attachment avoidance. Each item was rated on a 7-point rating scale, with responses ranging from 1 (*strongly disagree*) to 7 (*strongly agree*). Responses to the items were averaged to obtain composite scores for each subscale. Cronbach’s alpha was .96 for the anxiety subscale and .93 for the avoidance subscale.

*Television figure qualities.* The qualities of participants’ favorite television figures were assessed with a 16-item instrument loosely derived from the Sixteen Personality Factor (16PF) Questionnaire from Cattell (1957, 1973). Participants were presented with a list of 16 descriptors, with each descriptor corresponding to one of the 16 personality traits from the 16PF Questionnaire. The specific descriptors were warmth, reasoning, emotional stability, sensitivity, dominance, liveliness, rule-consciousness, social boldness, vigilance, abstractedness, privateness, apprehension, openness to change, self-reliance, perfectionism, and tension. Brief descriptions of each trait accompanied the descriptors (see Cattell & Mead, 2008; Conn & Rieke, 1994). Participants were asked to rate how well the descriptors fit the personality of their favorite television figure. Each item was rated on a 7-point rating scale, with responses ranging from 1 (*strongly disagree*) to 7 (*strongly agree*).

### Procedure

Participants were redirected from MTurk to the Qualtrics online survey platform, which hosted the study materials. After completing an informed consent form, participants filled out the demographic questionnaire and the attachment scale. Next, they were asked to write down the name of their favorite television figure, either a fictional character (for instance, a character from a drama or sitcom) or a nonfictional personality (for instance, a news anchor or talk show host). They were also asked to write down the name of the television program that their favorite television figure appears on. Finally, they filled out the instrument assessing their favorite television figure’s qualities. Once data collection was complete, data were entered into SPSS Statistics (Version 26.0) for statistical analysis.

## Results

Participants selected a wide variety of television figures as their favorites. The most frequently selected figures were Jon Snow (played by Kit Harington) from *Game of Thrones* (*N* = 6), Sheldon Cooper (played by Jim Parsons) from *The Big Bang Theory* (*N* = 4), Rachel Green (played by Jennifer Aniston) from *Friends* (N = 4), and Jean-Luc Picard (played by Patrick Stewart) from *Star Trek: The Next Generation* (*N* = 4). Overall, fictional characters (*N* = 147) were more common than nonfictional personalities (*N* = 53).

Pearson correlations were used to assess the bivariate relationships between attachment anxiety and each of the television figure descriptors, as well as attachment avoidance and the television figure descriptors. Due to the number of statistical tests being performed within each set of correlations, the alpha level of .05 was adjusted using the Holm-Bonferroni method (Holm, 1979). The Holm-Bonferroni method involves ranking the significance values for each statistical test from smallest to largest and evaluating those values using steadily less stringent criteria for significance. It provides protection against Type I error, but it is not as conservative as a standard Bonferroni correction.

Results showed significant positive associations between attachment anxiety and two television figure descriptors: apprehension, *r*(198) = .33, *p* < .001, and tension, *r*(198) = .27, *p* < .001. There were also significant negative associations between attachment anxiety and two television figure descriptors: reasoning, *r*(198) = −.25, *p* < .001, and self-reliance, *r*(198) = −.21, *p* = .003. In general, participants with higher levels of attachment anxiety reported favorite television figures that are more apprehensive and tense, with poorer reasoning and lower levels of self-reliance. No significant associations between attachment avoidance and the television figure descriptors were found.

An exploratory analysis was performed to assess whether the correlational results persisted after accounting for the fictional or nonfictional nature of the television figures. For this analysis, the correlation analysis was repeated using partial correlations with fictional/nonfictional status as the control variable. Once again, results showed that attachment anxiety was significantly related to apprehension, *pr*(197) = .33, *p* < .001; tension, *pr*(197) = .27, *p* < .001; reasoning, *pr*(197) = −.25, *p* < .001; and self-reliance, *pr*(197) = −.21, *p* = .002. No significant associations between attachment avoidance and the television figure descriptors were found.

A canonical correlation analysis was used to gain a more comprehensive understanding of how the attachment dimensions were related to the television figure descriptors. Canonical correlation is a dimension reduction technique that assesses the relationship between two sets of variables. Attachment anxiety, attachment avoidance, and the interaction between anxiety and avoidance formed one set of variables. The television figure descriptors formed the second set of variables. The canonical correlation analysis yielded three functions with canonical correlations of .57, .33, and .28, respectively. The full model across all three functions was statistically significant, *F*(48, 539.13) = 2.44, *p* < .001 (Wilks’s λ = .56). However, peel off testing showed that the model was no longer significant after removing the first function, *F*(30,364) = 1.25, *p* = .176. In other words, the first function accounted for the significant relationship between the two sets of variables.

Table 1 presents the standardized canonical correlation coefficients and canonical loadings for the first function. To facilitate analysis of results, a threshold of .39 was adopted for interpretation of the canonical loadings (representing ∼15% shared variance between the variable and the canonical variate). This value was selected as a compromise between a more liberal threshold of .32 (∼10% shared variance) and a more conservative threshold of .45 (∼20% shared variance; Tabachnick & Fidell, 2013). With respect to the attachment variables, the first function reflected high levels of attachment anxiety. Attachment avoidance and the interaction between anxiety and avoidance contributed comparatively little to the canonical variate. With respect to the television figure descriptors, the first function reflected high levels of apprehension, tension, and rule consciousness, as well as low levels of reasoning. The remaining descriptors contributed comparatively little to the canonical variate. Taken together, the results of the canonical correlation analysis indicate that individuals with higher levels of attachment anxiety are drawn to television figures that are more apprehensive, tense, and rule conscious, with poorer reasoning.

**Table 1.** Standardized canonical correlation coefficients and canonical loadings for function 1.

Variable	Standardized coefficient	Canonical loading
Set 1		
Attachment anxiety	0.87	.90*
Attachment avoidance	0.21	.24
Anxiety × avoidance	0.53	.32
Set 2		
Warmth	−0.07	.10
Reasoning	−0.48	−.41*
Emotional stability	−0.01	−.01
Sensitivity	0.11	.32
Dominance	−0.08	.13
Liveliness	−0.04	.10
Rule consciousness	0.39	.44*
Social boldness	0.33	.22
Vigilance	−0.03	.16
Abstractedness	0.04	.23
Privateness	0.08	.23
Apprehension	0.33	.67*
Openness to change	0.25	.13
Self-reliance	−0.19	−.24
Perfectionism	0.15	.18
Tension	0.36	.53*

*Note.* Canonical loadings above .39 (∼15% shared variance with the canonical variate) are marked with a * for emphasis.

## Discussion

Past research has shown that anxiously attached individuals tend to have stronger parasocial relationships than people with other attachment orientations. Researchers have suggested that anxiously attached individuals see their favorite media figures as surrogate attachment figures that offer a greater degree of safety and security than their real-life attachment figures. The results of the current study cast doubt on this notion. Participants with higher levels of attachment anxiety tended to report favorite television figures that themselves exhibited higher levels of anxiety and insecurity. It seems unlikely that anxiously attached individuals would derive feelings of safety and security from such figures, particularly when there are alternative television figures (perhaps even on the same program) that could more effectively provide such feelings. Previous studies from the parasocial relationship literature have shown that people are often drawn to media figures that remind them of themselves (Eyal & Rubin, 2003; Moyer-Gusé & Nabi, 2010). This finding can help to explain why anxiously attached individuals gravitate toward media figures with anxious or insecure qualities. Unfortunately, it does not explain why they are inclined to form stronger parasocial relationships than other people.

To account for the link between attachment anxiety and parasocial relationship strength, researchers may need to consider other perspectives. For instance, it is possible that anxiously attached individuals do form attachment bonds with their favorite media figures, but the nature of these bonds might not be as secure as previously thought. In this case, their media attachments would mirror their real-life attachments to a certain degree. Researchers should also consider the possibility that anxiously attached individuals do not see their favorite media figures as attachment figures at all, as there are other potential reasons why these individuals might develop strong parasocial connections. For instance, studies have shown that anxiously attached individuals tend to have a higher need for belonging and affiliation than people with other attachment orientations (Chen et al., 2015; Joyce, 2014). Perhaps these individuals view media figures as friends or companions that complement their real social contacts and provide an additional route for achieving feelings of interpersonal connectedness. Of course, these and other possibilities remain speculative for the time being.

Although the statistical analysis for this study revealed a number of significant results for attachment anxiety, no comparable results were found for attachment avoidance or the interaction between anxiety and avoidance. This outcome is not particularly surprising, as past research from the parasocial relationship literature has failed to show a consistent link between attachment avoidance and people’s bonds with television figures. People with high levels of attachment avoidance clearly use television and have favorite television figures. However, attachment avoidance may not be related to preferences for specific types of television figures, regardless of whether it is considered in isolation or in conjunction with attachment anxiety (at the very least, such preferences were not evident in this study). Attachment anxiety by itself seems to be the best indicator of preferred television figure qualities.

### Limitations and future directions

There were some important limitations to this research. Ratings of television figure qualities were used to assess whether anxiously attached individuals see their favorite television figures as safe and secure attachment figures. However, cognitive, affective, and behavioral responses to these figures would provide an alternative and potentially more valid means to test this possibility. Although anxiously attached individuals are drawn to relatively anxious and insecure television figures, it is possible (unlikely though it may seem) that they derive a sense of attachment security from such figures. Future researchers should assess people’s responses to media figures to better determine how anxiously attached individuals view these figures. Researchers should also consider alternate, nonattachment reasons for why anxiously attached individuals might be drawn to such figures, such as the satisfaction of belongingness needs.

The qualities of participants’ favorite television figures were assessed using single items representing each of Cattell’s 16 personality factors. These collective items could be administered fairly quickly (the full version of Cattell’s 16PF Questionnaire contains 185 items in its current form) while also providing a nuanced view of people’s favorite television figures. However, these single items likely lacked the sensitivity and reliability of multi-item measures. Several studies have shown that the performance of single-item measures is often comparable to that of multi-item measures (e.g., Bergkvist & Rossiter, 2007; Gardner et al., 1998; Killgore, 1999). Regardless, using multiple items to assess each personality factor may have resulted in a different pattern of results than was seen in the current study. In the future, researchers should use a larger assessment instrument with more established psychometric properties.

## Conclusion

The results of this study challenge the assertion that anxiously attached individuals see their favorite media figures as surrogate attachment figures that compensate for deficiencies in real-life attachment security. Although unexpected, these results open some interesting avenues for future research in this area.

## Note

1 Exploratory analyses failed to show differences in preferred television figure qualities according to gender, age, or race/ethnicity, and so these variables are not addressed in further analyses.

## Notes

**Author Biographies:** **A. Luke MacNeill** was a graduate student in the Department of Psychology at the University of New Brunswick in Saint John. He is currently a postdoctoral fellow at the same university. **Enrico DiTommaso** is a professor in the Department of Psychology at the University of New Brunswick in Saint John.

## References

[bibr1-00332941211002142] Bartholomew K. Horowitz L. M. (1991). Attachment styles among young adults: A test of a four-category model. Journal of Personality and Social Psychology, 61(2), 226–244. 10.1037/0022-3514.61.2.226 1920064

[bibr2-00332941211002142] Bergkvist L. Rossiter J. R. (2007). The predictive validity of multiple-item versus single-item measures of the same constructs. Journal of Marketing Research, 44(2), 175–184. 10.1509/jmkr.44.2.175

[bibr3-00332941211002142] Bowlby J. (1969). Attachment and loss, volume 1: Attachment. Basic Books.

[bibr4-00332941211002142] Bowlby J. (1973). Attachment and loss, volume 2: Separation. Basic Books.

[bibr5-00332941211002142] Brennan K. A. Clark C. L. Shaver P. R. (1998). Self-report measurement of adult attachment: An integrative overview. In Simpson J. A. Rholes W. S. (Eds.), Attachment theory and close relationships (pp. 46–76). The Guilford Press.

[bibr6-00332941211002142] Cattell R. B. (1957). Personality and motivation structure and measurement. World Book.

[bibr7-00332941211002142] Cattell R. B. (1973). Personality and mood by questionnaire. Jossey-Bass.

[bibr8-00332941211002142] Cattell H. E. P. Mead A. D. (2008). The sixteen personality factor questionnaire (16PF). In Boyle G. J. Matthews G. Saklofske D. H. (Eds.), The Sage handbook of personality theory and assessment, volume 2: Personality measurement and testing (pp. 135–159). Sage.

[bibr9-00332941211002142] Chen C. Hewitt P. L. Flett G. L. (2015). Preoccupied attachment, need to belong, shame, and interpersonal perfectionism: An investigation of the perfectionism social disconnection model. Personality and Individual Differences, 76, 177–182. 10.1016/j.paid.2014.12.001

[bibr10-00332941211002142] Cohen J. (2004). Parasocial break-up from favorite television characters: The role of attachment styles and relationship intensity. Journal of Social and Personal Relationships, 21(2), 187–202. 10.1177/0265407504041374

[bibr11-00332941211002142] Cole T. Leets L. (1999). Attachment styles and intimate television viewing: Insecurely forming relationships in a parasocial way. Journal of Social and Personal Relationships, 16(4), 495–511. 10.1177/0265407599164005

[bibr12-00332941211002142] Conn S. R. Rieke M. L. (1994). The 16PF fifth edition technical manual. Institute for Personality and Ability Testing.

[bibr13-00332941211002142] Derrick J. L. Gabriel S. Hugenberg K. (2009). Social surrogacy: How favored television programs provide the experience of belonging. Journal of Experimental Social Psychology, 45(2), 352–362. 10.1016/j.jesp.2008.12.003

[bibr14-00332941211002142] Eyal K. Dailey R. M. (2012). Examining relational maintenance in parasocial relationships. Mass Communication and Society, 15(5), 758–781. 10.1080/15205436.2011.616276

[bibr15-00332941211002142] Eyal K. Rubin A. M. (2003). Viewer aggression and homophily, identification, and parasocial relationships with television characters. Journal of Broadcasting & Electronic Media, 47(1), 77–98. 10.1207/s15506878jobem4701_5

[bibr16-00332941211002142] Gardner D. G. Cummings L. L. Dunham R. B. Pierce J. L. (1998). Single-item versus multiple-item measurement scales: An empirical comparison. Educational and Psychological Measurement, 58(6), 898–915.

[bibr17-00332941211002142] Gardner W. L. Pickett C. L. Knowles M. (2005). Social snacking and shielding: Using social symbols, selves, and surrogates in the service of belonging needs. In. Williams K. D. Forgas J. P. von Hippel W. (Eds.), The social outcast: Ostracism, social exclusion, rejection, and bullying (pp. 227–241). Psychology Press.

[bibr18-00332941211002142] Greenwood D. N. Pietromonaco P. R. Long C. R. (2008). Young women's attachment style and interpersonal engagement with female TV stars. Journal of Social and Personal Relationships, 25(3), 387–407. 10.1177/0265407507087964

[bibr19-00332941211002142] Hartmann T. (2016). Parasocial interaction, parasocial relationships, and well-being. In Reinecke L. Oliver M. B. (Eds.), The Routledge handbook of media use and well-being: International perspectives on theory and research on positive media effects (pp. 131–144). Routledge.

[bibr20-00332941211002142] Holm S. (1979). A simple sequentially rejective multiple test procedure. Scandinavian Journal of Statistics, 6(2), 65–70.

[bibr21-00332941211002142] Jin B. Kim J. (2015). Television drama viewing and romantic beliefs: Considering parasocial interaction and attachment style. International Journal of Humanities and Social Science, 5(10), 51–60.

[bibr22-00332941211002142] Joyce B. A. (2014). *Matters of the heart? The role of risk regulation, attachment style, self-monitoring and self-esteem in romantic relationships* (Unpublished doctoral dissertation). University of South Carolina – Aiken.

[bibr23-00332941211002142] Kassing J. W. Sanderson J. (2009). “Is this a church? Such a big bunch of believers around here!”: Fan expressions of social support on floydlandis.com. Journal of Communication Studies, 1(special ed), 308–329.

[bibr24-00332941211002142] Killgore W. D. S. (1999). The visual analogue mood scale: Can a single-item scale accurately classify depressive mood state? Psychological Reports, 85(3 Pt 2), 1238–1243. 10.2466/PR0.85.7.1238-1243 10710979

[bibr25-00332941211002142] Mikulincer M. Shaver P. R. (2007). Attachment in adulthood: Structure, dynamics, and change. Guilford Press.

[bibr26-00332941211002142] Moyer-Gusé E. Nabi R. L. (2010). Explaining the effects of narrative in an entertainment television program: Overcoming resistance to persuasion. Human Communication Research, 36(1), 26–52. 10.1111/j.1468-2958.2009.01367.x

[bibr27-00332941211002142] Rosaen S. F. Dibble J. L. (2016). Clarifying the role of attachment and social compensation on parasocial relationships with television characters. Communication Studies, 67(2), 147–162. 10.1080/10510974.2015.1121898

[bibr28-00332941211002142] Rubin R. B. McHugh M. P. (1987). Development of parasocial interaction relationships. Journal of Broadcasting & Electronic Media, 31(3), 279–292. 10.1080/08838158709386664

[bibr29-00332941211002142] Schramm H. Hartmann T. (2008). The PSI-Process scales: A new measure to assess the intensity and breadth of parasocial processes. Communications, 33(4), 385–401. 10.1515/COMM.2008.025

[bibr30-00332941211002142] Tabachnick B. G. Fidell L. S. (2013). Using multivariate statistics (6th ed.). Pearson.

[bibr31-00332941211002142] Theran S. A. Newberg E. M. Gleason T. R. (2010). Adolescent girls’ parasocial interactions with media figures. The Journal of Genetic Psychology, 171(3), 270–277. 10.1080/00221325.2010.483700 20836434

[bibr32-00332941211002142] Tsao J. (1996). Compensatory media use: An exploration of two paradigms. Communication Studies, 47(1-2), 89–109. 10.1080/10510979609368466

